# Acute Esophageal Necrosis in an Alcoholic after Successful Resuscitation from Cardiac Arrest

**DOI:** 10.1155/2017/5092906

**Published:** 2017-06-19

**Authors:** Amish Shah, Viveksandeep Thoguluva Chandreskar, Ravi Doobay, Arundeep Kahlon, Ioana Amzuta

**Affiliations:** ^1^Internal Medicine Department, State University of New York Upstate Medical University, 750 E Adams St, Syracuse, NY 13210, USA; ^2^Gastroenterology Department, State University of New York Upstate Medical University, 750 E Adams St, Syracuse, NY 13210, USA; ^3^Pulmonary/Critical Care Department, State University of New York Upstate Medical University, 750 E Adams St, Syracuse, NY 13210, USA

## Abstract

**Introduction:**

We present a patient who presented to the ICU after successful resuscitation from cardiac arrest who was subsequently diagnosed with AEN.

**Case Presentation:**

A 66-year-old female presented after cardiac arrest in which return of spontaneous circulation was achieved within 7 minutes after the initiation of CPR. She was intubated on the scene and found to have coffee ground emesis in her bathroom when found unresponsive. Due to the hemodynamically significant GI bleed, patient was started on IV proton pump inhibitor, octreotide, and levophed. Subsequent endoscopy showed diffuse severe mucosal changes characterized by blackness, erythema, friability, granularity, inflammation, and decreased vascular pattern in the middle third of the esophagus and in the lower third of the esophagus.

**Discussion:**

AEN is a rare syndrome with a prevalence ranging from 0.001 to 0.2% of EGD. This patient is especially rare as this patient was female and had AEN in the middle esophagus along with lower esophagus. The pathophysiology in this patient is hypothesized that she had cardiac arrest secondary to acute upper GI hemorrhage. Subsequent low-flow state (cardiac arrest) in addition to being in the setting of severe alcohol abuse led to esophageal necrosis.

## 1. Introduction

Acute esophageal necrosis (AEN) is a rare syndrome characterized by diffuse circumferential black appearance of esophageal mucosa that affects the distal esophagus on endoscopy [1]. The prevalence of this syndrome ranges from 0.001 to 0.2% of endoscopies [2]. There is a high mortality rate of 32% found in this population [3]. We are presenting a unique case of a patient who presented to the ICU after successful resuscitation from cardiac arrest who was subsequently found to have been diagnosed with AEN during endoscopy.

## 2. Case Presentation

A 66-year-old female with unknown past medical history except for alcohol abuse (daily use of 1-2 pints of hard liquor) and other illicit drugs (history of PCP, benzodiazepine, and methadone abuse) presented as a transfer from an outside hospital after cardiac arrest. Return of spontaneous circulation was achieved within 7 minutes after the initiation of CPR. She was noted to have coffee ground emesis in her bathroom when found unresponsive and was intubated on the scene.

On admission to the ICU, she was afebrile with a temperature of 97.7°F, tachycardic with a pulse of 115/min, tachypneic with a respiratory rate of 22/min, and was hypotensive (a low blood pressure as low as 75/48). She required pressor support with norepinephrine. Additionally, she was started on intravenous pantoprazole and octreotide for concerns of an ongoing GI bleed with the source unclear.

**Figure 1:**
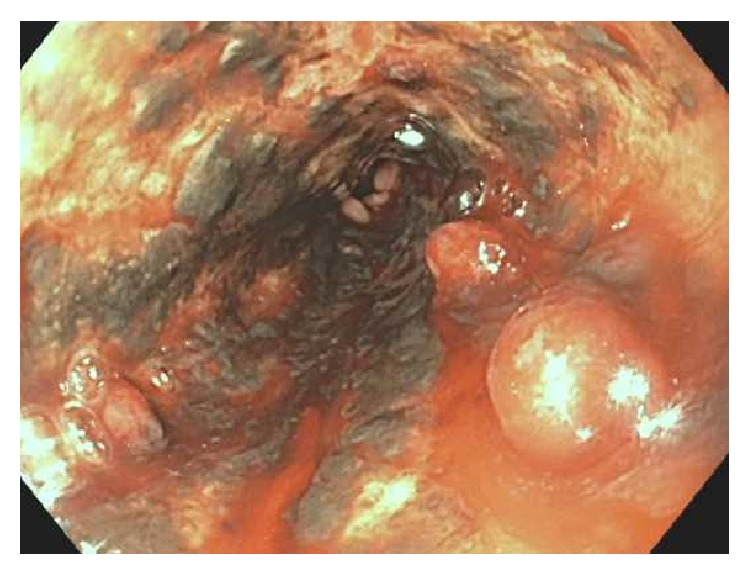
Middle esophagus showing discoloration, erythema, friability (with contact bleeding), granularity, hemorrhagic appearance, inflammation, nodularity, altered texture, and a decreased vascular pattern.

**Figure 2:**
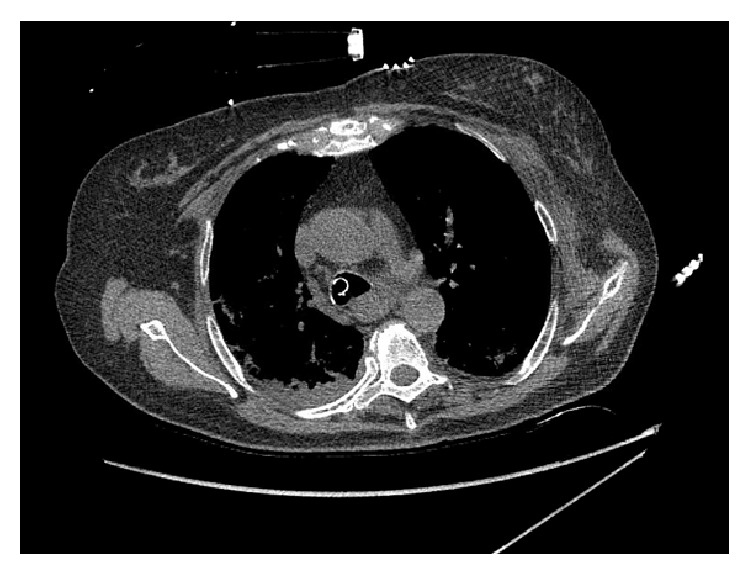
Moderate size hiatal hernia, esophageal enlargement diffusely with hyperdense material noted centrally within the stomach and distal esophagus. Given the history, this is concerning for gastric/distal esophageal hemorrhage but remains nonspecific. Acute sternal fracture was also found.

Gastroenterology service was consulted on admission. An emergent upper GI endoscopy was performed which revealed diffuse severe mucosal changes characterized by black interspersed with erythematous mucosa which was friable and granular and had decreased vascular pattern in the middle third and lower third of the esophagus concerning for ischemia (Figure 1). No esophageal varices were seen and stomach appeared normal. CT thorax (Figure 2) revealed diffusely enlarged esophagus with hyperdense material noted in the distal esophagus, fundus of the stomach, concerning for hemorrhage into the stomach. There was also an acute sternal fracture with multiple rib fractures likely secondary to cardiopulmonary resuscitation. Patient was continued on supportive care with intravenous fluids, pantoprazole, octreotide, and blood transfusions. Despite aggressive resuscitation, the patient was diagnosed with anoxic brain injury. She received comfort and care by the family and eventually passed away within few days. Autopsy was not performed due to family's refusal.

## 3. Discussion

AEN also known as black esophagus is a rare syndrome with a prevalence ranging from 0.001 to 0.2% of EGD [2] that was first described by Goldenberg et al. [4]. With mortality rate as high as 32% in this population, patients often go undiagnosed until an autopsy is performed [3, 5, 6].

There are multiple comorbidities associated with AEN. Among them, those placing a patient at highest risk of developing AEN include diabetes mellitus, malignancy, hypertension, alcohol abuse, and coronary artery disease.

There is also a gender difference in the incidence of AEN. Men are four times more commonly affected [7].

Most commonly, patients will present with hematemesis and/or melena [8]. Additionally, they will have other symptoms suggestive of sepsis including lactic acidosis, tachycardia, and hypotension.

The accepted hypothesis behind the pathophysiology is a two-hit hypothesis [9, 10]. There is an initial event that predisposes the patient to subsequent injury. We hypothesize that the initial event of chronic alcohol abuse leads to decreased gastric defenses and increased acidic gastric contents in the esophagus. Her acute upper GI hemorrhage in this setting of severe alcohol abuse (first hit) likely led to cardiac arrest due to hypovolemia. This low-flow state (second hit) may have been exacerbated by acute sternal fracture due to chest compression during cardiopulmonary resuscitation. The middle esophageal blood supply is derived from the bronchial arteries, right third or fourth intercostal arteries, and numerous small esophageal arteries off the descending aorta. In addition to the cardiac arrest, the acute hemorrhage found on the CT thorax along with acute sternal fracture, it is hypothesized that the cardiopulmonary resuscitation and sternal fracture may have severed these bronchial and intercostal arteries leading to decreased blood supply to the middle esophagus. The influx of gastric contents in the esophagus where gastric defenses were already compromised due to alcohol abuse and low-flow state leads to the necrosis seen in our patient. Our patient, retrospectively, fits this hypothesis.

Endoscopy is the gold standard in diagnosing AEN. Findings on CT thorax are largely nonspecific and, therefore, endoscopy is always necessary to diagnose AEN. As seen in the CT thorax for our patient, hemorrhage was noted as hyperdense material, but these findings were nonspecific for AEN.

The treatments for patients with AEN are aggressive resuscitation and time. There is no reversal agent or surgery to reverse the damage. If caught early enough and resuscitation is provided via IV fluids and blood transfusions, AEN can be reversed [1].

Despite aggressive resuscitation, mortality rate is still high at 32% for this condition.

Our patient's presentation of AEN was unique in two distinct ways; she was female (it is four times more common in males) and necrosis was found in the middle and lower esophagus. Normally, only the distal third esophagus is found to be necrotic as its blood supply is comparable to a watershed area [3].

## 4. Conclusion

Only one major retrospective review regarding AEN has been published which reviewed 88 documented case reports from 1965 to 2006, which was highlighted in a much-cited article by Gurvits et al. in 2007 [8]. Further research needs to be conducted as many statistics and conclusions have been made based off of the 88 patients found in that review article. As almost 10 years have passed since that review article, and being an extremely rare condition, further research must be done to help triage patients who may benefit from a high suspicion of acute esophageal necrosis to decrease the mortality and morbidity from this condition.
